# In-Hospital Rehabilitation for Frailty Management May Improve Quality of Life in Patients Receiving Maintenance Dialysis: A Retrospective Observational Study

**DOI:** 10.7759/cureus.109144

**Published:** 2026-05-18

**Authors:** Toshie Yonemochi, Kenji Ina, Hiroko Ina, Atsushi Morizane, Megumi Kabeya, Miki Nagasaka, Ayami Sakaguchi, Airi Taniguchi, Shoudai Nishiyama, Yoshihiro Ohta

**Affiliations:** 1 Department of Rehabilitation, Shinseikai Daiichi Hospital, Nagoya, JPN; 2 Department of Geriatrics, Shinseikai Daiichi Hospital, Nagoya, JPN; 3 Department of Nursing, Koujin Hospital, Nagoya, JPN; 4 Department of Clinical Engineering, HOSPY Renal Dialysis Division, Nagoya, JPN; 5 Department of Pharmacy, Nagoya Memorial Hospital, Nagoya, JPN; 6 Department of Nephrology, Shinseikai Daiichi Hospital, Nagoya, JPN

**Keywords:** frailty measures hospitalization, geriatric depression scale" (gds-15), japanese rehabilitation, kdqol-36™, maintenance hemodialysis, retrospective observational study

## Abstract

Background

Quality of life (QOL) is defined as an individual’s perception of his or her position in life in the context of culture and value systems. Previous studies have shown that hemodialysis markedly affects patients’ QOL, and health-related QOL has been increasingly recognized as an important medical outcome in patients undergoing maintenance hemodialysis (MHD).

Methods

A two-week hospitalization program for patients undergoing MHD was introduced in 2023 to evaluate frailty, along with comprehensive geriatric assessment (CGA) and evaluation of motor and oral cavity functions. During this program, QOL was assessed using the Kidney Disease Quality of Life-36 (KDQOL-36) questionnaire, and physical exercise was instructed according to the Japanese Renal Rehabilitation Guideline. After the incidence of each item was determined, the associations between these factors were retrospectively analyzed to identify the specific factors affecting QOL.

Results

QOL, as determined using the physical component summary (PCS) and mental component summary (MCS), decreased in more than 40% of the enrolled patients. Motor function was often impaired in patients undergoing MHD. In univariate analyses, the PCS score was strongly correlated with seven variables, including lower-limb strength/body weight (%), gait speed (5 m), the 6-minute walk test (6MWT), the Short Physical Performance Battery (SPPB), the Eating Assessment Tool-10 (EAT-10) score, the Tokyo Metropolitan Institute of Gerontology (TMIG) Index of Competence, and the Geriatric Depression Scale-15 (GDS-15) score, but not with age, sex, dialysis duration, primary disease, comorbidities, or nutritional status. For the MCS, only gait speed (m/s) and the GDS-15 score were significantly correlated. In the multivariate analysis, the PCS score remained independently associated with the 6MWT (β = 0.0727, p = 0.0109) and GDS-15 score (β = -2.14, p = 0.0068), whereas the GDS-15 score was the sole independent determinant of the MCS score (β = -2.81, p = 0.0047).

Conclusions

A decline in physical function was commonly observed in patients undergoing MHD. Among the KDQOL components, the PCS score worsened with decreasing motor function, especially walking ability. Moreover, depressive symptoms appeared to significantly impact QOL in this population. These findings suggest that physical exercise guidance provided to patients according to the Japanese Renal Rehabilitation Guideline, referred to as Japanese rehabilitation, may be helpful for enhancing the overall QOL of patients undergoing MHD through the dual action of improving walking ability and alleviating depressive symptoms.

## Introduction

In the literature, the term quality of life (QOL) is also often referred to as “well-being.” The WHO defines QOL as individuals’ perception of their position in life within the context of the culture and value systems in which they live and in relation to their goals, expectations, standards, and concerns [1]. Patients undergoing hemodialysis have a greater risk of hospitalization, heart problems, and death [2], which leads to reduced QOL. Studies have shown that dialysis affects the QOL of patients [3,4], and QOL has become a critical metric for evaluating the well-being of individuals undergoing hemodialysis. Health-related QOL has been increasingly recognized as an important medical outcome in this population [5]. Various tools are available to measure QOL, including disease-specific tools. The Kidney Disease Quality of Life Short Form (KDQOL-SF) was developed by RAND to assess QOL in patients with chronic kidney disease (CKD) [6,7].

Shinseikai Daiichi Hospital, located in the eastern part of Nagoya City, has 144 hospital beds and 100 dialysis beds. Approximately 1,300 patients undergo hemodialysis in our group. Given that the average age of these patients is 73.3 years, instrumental activities of daily living (IADLs) are frequently reduced, and a decline in muscle strength is commonly observed [8].

We evaluated the QOL of patients undergoing maintenance hemodialysis (MHD) using the Kidney Disease Quality of Life-36 (KDQOL-36™) questionnaire [9] and investigated the associations among motor features, oral cavity function, findings obtained via a comprehensive geriatric assessment (CGA) [10], and nutritional assessment to identify the factors that impact QOL in these patients.

## Materials and methods

Study design and participants

**Figure 1:**
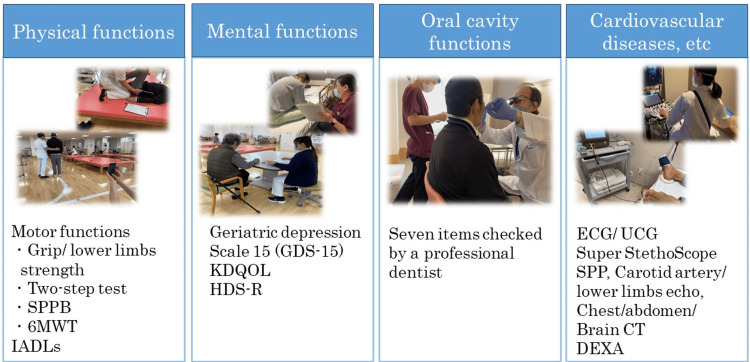
Hospitalization for frailty assessment. GDS-15: Geriatric Depression Scale-15; KDQOL: Kidney Disease Quality of Life; HDS-R: Hasegawa’s Dementia Scale-Revised; SPPB: Short Physical Performance Battery; 6MWT: 6-Minute Walk Test; IADLs: Instrumental Activities of Daily Living; UCG: Ultrasound Cardiography; SPP: Skin Perfusion Pressure; DEXA: Dual-Energy X-ray Absorptiometry.

In October 2023, we initiated a two-week hospitalization program for patients undergoing MHD (frailty measures hospitalization) to evaluate physical and mental frailty and investigate the risk of cardiovascular diseases and other organic diseases (Figure 1). During the two-week frailty measures hospitalization program, our patients receive instruction and supervision by a physical therapist (PT) according to the Japanese Renal Rehabilitation Guideline. The patients are instructed to exercise for one hour at Borg 11-13 five times a week, comprising stretching at first, resistance training focused on the lower extremities, aerobic exercise such as walking and the use of an ergometer, and balance training. The specific program for each patient is determined by the PT in charge, based on his or her motor function results assessed upon admission. The criteria for frailty measures hospitalization included patients undergoing hemodialysis who were willing to participate in the program and who could walk independently. Patients undergoing MHD who participated in this program between October 2023 and September 2025 were successively recruited.

QOL assessment

The participants completed the Kidney Disease Quality of Life Short Form version 1.3 (KDQOL-36™), which is a widely used tool for assessing health-related QOL [6,7,11]. This questionnaire is designed to provide a comprehensive view of a patient’s overall QOL and can be used to monitor changes over time [9]. We obtained permission from Qualitest (Kyoto, Japan) and followed the scoring rules provided in the KDQOL-SF™ user manual. The precoded items were transformed into a 0-100 scale, with higher scores representing better QOL. The KDQOL-36™ consists of 36 items describing the perception of health status during the past four weeks, including symptoms and problems, the effects of kidney disease on daily life, the burden of kidney disease, work status, cognitive function, the quality of social interaction, sexual function, sleep, social support, dialysis staff encouragement, patient satisfaction, physical functioning, physical role, pain, general health, emotional well-being, emotional role, social functioning, and energy/fatigue. The physical component summary score (PCS score = physical functioning + role limitations due to physical health + pain + general health) and the mental component summary score (MCS score = energy + social functioning + role limitations due to emotional problems + emotional well-being) were subsequently determined.

Comprehensive geriatric, motor function, oral frailty, and nutritional assessment

The CGA was performed upon admission using the Tokyo Metropolitan Institute of Gerontology (TMIG) index of competence, the Geriatric Depression Scale-15 (GDS-15), the vitality index, and Hasegawa’s Dementia Scale-Revised (HDS-R) [10]. Motor function was evaluated by measuring grip strength, leg muscle strength, gait speed (5 m), the 6-minute walk test (6MWT), sit-to-stand-5 (SS-5), and the Short Physical Performance Battery (SPPB). In addition, a two-step test was performed to determine the degree of locomotive syndrome (severe: < 0.9, mild: 0.9 or above and below 1.3, robust: > 1.3). Upon admission, seven oral cavity function items were assessed by a professional dentist. The 10-item Eating Assessment Tool (EAT-10) is a self-administered, symptom-specific outcome instrument for dysphagia [12]. In our analysis, oral frailty was defined as a poor result in > 3 of the 7 measures [8]. The BMI and the Global Leadership Initiative on Malnutrition (GLIM) criteria were used for nutritional assessment [13].

Statistical analysis

This was a retrospective observational study. The data were analyzed using descriptive statistics. The results of the stratified analysis were compared between the two groups using Fisher’s exact test or the Mann-Whitney U test. To examine the associations between continuous variables and QOL measures, Pearson’s correlation coefficient (r) was used. Variables with p < 0.10 in univariate analyses were entered into multivariate linear regression models. Stepwise variable selection based on the Akaike information criterion (AIC) was employed to identify independent predictors. Multicollinearity was assessed using variance inflation factors (VIFs), with a VIF of < 5 considered acceptable. In addition, we determined the coefficient of determination (R²) to evaluate the explanatory power of our multivariate models. All p-values were two-sided, and p-values of 0.05 or less were considered to indicate statistical significance. Statistical analyses were performed using EZR (Saitama Medical Center, Jichi Medical University, Saitama, Japan), a graphical user interface for R (R Foundation for Statistical Computing, Vienna, Austria) [14]. This study was approved by the Ethics Committee of Shinseikai Daiichi Hospital (S2023-#011; approved on February 3, 2024).

## Results

Baseline characteristics and KDQOL

Seventy-seven patients undergoing MHD participated in the frailty measures hospitalization program between October 2023 and September 2025. Among these consecutive patients, 70 individuals who participated for the first time were enrolled in the present retrospective observational study. The patient demographic characteristics are listed in Table 1.

**Table 1:** Demographic characteristics of the patients enrolled in the present study.

	Total	Male	Female
Sex	70	52	18
Age (years), median (range)	76.5 (36-90)	76 (36-90)	78 (48-89)
Duration of hemodialysis (months), median (range)	7.0 (0-22)	6.5 (0-22)	8 (0-20)
Primary disease			
Diabetic kidney disease	40	31	9
Nephrosclerosis	15	12	3
Nephritis	7	5	2
Others	8	4	4
Comorbidities			
Cardiovascular diseases, including heart failure, history of coronary artery bypass grafting, and myocardial infarction	36	28	8
Neurological disorders, including stroke and a history of Parkinson’s disease	33	24	9
Musculoskeletal disorders, including fractures and a history of orthopedic surgery	15	11	4

Table 2 shows the QOL scores determined using the KDQOL-36™ questionnaire. Both the PCS and MCS scores were decreased in more than 40% of the enrolled patients, whereas the KDQOL Disease-Specific and Non-Health scores were infrequently impaired (median (range): KDQOL Disease-Specific, 74.32 (37.85-96.88); Non-Health, 74.97 (16.67-100); PCS, 50.94 (12.5-97.5); MCS, 69.25 (7.25-99)).

**Table 2:** KDQOL-36™ scores in patients undergoing MHD. MHD: Maintenance hemodialysis; KDQOL-36™: Kidney Disease Quality of Life-36; QOL: Quality of life; PCS: Physical Component Summary; MCS: Mental Component Summary.

KDQOL-36™	Median (range)	Cutoff value	Prevalence (%)
Disease-Specific	74.32 (37.85-96.88)	65.76	19.7
Non-Health	74.97 (16.67-100)	68.63	25.8
PCS	50.94 (12.5-97.5)	46.67	43.9
MCS	69.25 (7.25-99)	59.19	40.9

The TMIG index of competence was frequently decreased, whereas 12.8% of the enrolled patients had GDS-15 scores greater than 10 points, indicating depression (Table 3). Cognitive impairment, evaluated using the HDS-R, was observed in 7.1% of these patients undergoing MHD. With respect to motor function, grip strength was decreased in 45 patients (men: 34/52, 65.3%; women: 11/18, 61.1%). Leg muscle strength and the 6MWT were reduced to a similar degree as decreased grip strength (64.3% and 66.7%, respectively), whereas gait speed was reduced in 36.2% of patients, and the time required for the SS-5 was prolonged in 50.7%. In contrast, compared with the indices above, the SPPB was impaired more frequently (81.2%), and locomotive syndrome was evident in 95.6% of patients (Table 4). Although oral frailty was observed in more than 60% of patients, dysphagia assessed using the EAT-10 was observed in only 14% of patients. Poor nutritional status, as determined by the GLIM criteria, was observed in 9% of patients.

**Table 3:** Comprehensive geriatric assessment. TMIG index: Tokyo Metropolitan Institute of Gerontology Index of Competence; GDS-15: Geriatric Depression Scale-15; HDS-R: Hasegawa’s Dementia Scale-Revised.

Item	Cutoff value	Full/Normal (%)	Decreased/Impaired (%)
TMIG Index of Competence	13	15.4	84.6
Instrumental	5	46.2	53.8
Intellectual	4	43.6	56.4
Social	4	25.6	74.4
Vitality Index	10	69.2	30.8
GDS-15	≥10	87.2	12.8
HDS-R	≤20	92.9	7.1

**Table 4:** Physical function was decreased in patients undergoing MHD. MHD: Maintenance hemodialysis; 6MWT: 6-minute walk test; SS-5: Sit-to-stand-5; SPPB: Short Physical Performance Battery.

Physical function	Cutoff value	Mean	SD	Median (range)	Prevalence (%)
Grip strength, male	26 kg	23.87	4.77	24 (13-38)	65.3
Grip strength, female	18 kg	15.72	5.16	14.75 (6-26)	61.1
Leg muscle strength	0.4 kgf/kg	0.33	0.12	0.3 (0.13-0.65)	64.3
Gait speed	1.0 m/s	1.13	0.44	1.14 (0.14-2.38)	36.2
6MWT	300 m	244.22	126.06	244 (25-576)	66.7
SS-5	14.5 s	13.12	7.87	11.5 (0-47)	50.7
SPPB	12 points	8.71	2.95	9 (0-12)	81.2
Two-step test	1.3 cm/cm	0.74	0.34	0.78 (0-1.45)	95.6

Associations among KDQOL-36™ scores, motor performance, the CGA, oral frailty, and nutritional conditions

We then investigated the associations of KDQOL-36™ scores with motor features, oral cavity function, the CGA, and nutritional status to identify factors impacting QOL in patients undergoing MHD. In the univariate analyses, the PCS score was significantly correlated with seven variables: lower-limb strength/body weight (%), gait speed, the 6MWT score, SPPB score, EAT-10 score, TMIG index of competence, and GDS-15 score (Table 5). The PCS score was not significantly correlated with age, sex, duration of dialysis, primary disease, comorbidities, or nutritional parameters. Univariate analyses of the 16 variables revealed that only the 5-m walk speed (m/s) and GDS-15 score were significantly correlated with the MCS score (Table 6). In the multivariate linear regression analysis using stepwise AIC selection, the PCS score remained independently associated with the 6MWT (β = 0.0727, p = 0.0109) and GDS-15 score (β = -2.14, p = 0.0068), with moderate explanatory power (R² = 0.3995, adjusted R² = 0.345). In contrast, the GDS-15 score was identified as the sole independent determinant of the MCS score (β = -2.81, p = 0.0047), although the explanatory power of the model was modest (R² = 0.2065, adjusted R² = 0.1838). These findings indicate that, while depressive symptoms were consistently associated with both PCS and MCS scores, a substantial proportion of the variance in QOL remains unexplained, particularly for the mental component.

**Table 5:** Association between PCS and clinical variables: univariate Pearson correlation and multivariate AIC-based final model. PCS: Physical Component Summary; 6MWT: 6-minute walk test; SS-5: Sit-to-stand-5; SPPB: Short Physical Performance Battery; TMIG index: Tokyo Metropolitan Institute of Gerontology Index of Competence; GDS-15: Geriatric Depression Scale-15; HDS-R: Hasegawa’s Dementia Scale-Revised; AIC: Akaike Information Criterion.

Variable (unit)	Univariate statistic, r	Univariate p-value	Multivariate statistic, β (95% CI)	Multivariate p-value
Age (years)	0.00385	0.9755		
BMI (kg/m²)	0.0978	0.4347		
Dialysis duration (months)	-0.135	0.2812		
Grip strength (kg)	0.0686	0.5839		
Lower-limb strength/body weight (%)	0.255	0.0386		
Gait speed (m/s)	0.407	0.000763		
6MWT (m)	0.46	0.000116	0.0727 (0.018 to 0.127)	0.0109
SS-5 (s)	-0.229	0.0667		
SPPB (total score)	0.247	0.0474		
Locomotive syndrome (score)	0.231	0.0661		
Oral function, precision test (yes/no)	-0.0495	0.6933		
EAT-10 (score)	-0.327	0.00731		
TMIG index of competence (score)	0.353	0.0319		
GDS-15 (score)	-0.356	0.0306	-2.14 (-3.62 to -0.66)	0.0068
Vitality index (motivation)	0.167	0.325		
HDS-R (score)	0.0235	0.8515		

**Table 6:** Association between MCS and clinical variables: univariate Pearson correlation and multivariate AIC-based final model. MCS: Mental Component Summary; 6MWT: 6-minute walk test; SS-5: Sit-to-stand-5; SPPB: Short Physical Performance Battery; TMIG index: Tokyo Metropolitan Institute of Gerontology Index of Competence; GDS-15: Geriatric Depression Scale-15; HDS-R: Hasegawa’s Dementia Scale-Revised; AIC: Akaike Information Criterion.

Variable (unit)	Univariate statistic, r	Univariate p-value	Multivariate statistic, β (95% CI)	Multivariate p-value
Age (years)	0.206	0.0974		
BMI (kg/m²)	0.14	0.261		
Dialysis duration (years)	-0.0744	0.553		
Grip strength (kg)	-0.152	0.224		
Lower-limb strength/body weight (%)	0.0639	0.61		
Gait speed (m/s)	0.313	0.0112		
6MWT (m)	0.229	0.0663		
SS-5, sit-to-stand (s)	-0.118	0.349		
SPPB (total score)	0.225	0.0721		
Locomotive syndrome (score)	0.238	0.0581		
Oral function, precision test (yes/no)	0.224	0.0702		
EAT-10 (score)	-0.0972	0.438		
TMIG index of competence (score)	0.213	0.206		
GDS-15 (score)	-0.454	0.0047	-2.81 (-4.67 to -0.95)	0.0047
Vitality index (motivation)	0.169	0.318		
HDS-R (score)	-0.0873	0.486		

## Discussion

Patient-reported outcomes, including health-related QOL and symptoms, are often identified by patients undergoing MHD as being more important than clinical outcomes such as survival. We performed a retrospective observational study to determine the factors associated with QOL in patients undergoing MHD using the KDQOL-36™. With respect to the KDQOL-36™ results, compared with the disease-specific and nonhealth domains, the PCS and MCS scores were more closely associated with QOL in patients undergoing MHD. A previous report showed that age, dialysis duration, hemoglobin levels, depression severity, and social support were key predictors of QOL across the KDQOL-36™ subscales. In contrast, we found that the PCS score was significantly associated with walking ability, as assessed by the 6MWT, and depressive tendencies, as assessed by the GDS-15, but not with age, sex, duration of dialysis, primary disease, comorbidities, or nutritional status. The explanatory power of the PCS model was moderate (adjusted R² = 0.345), suggesting that both walking ability and depressive symptoms contribute meaningfully, but not exclusively, to physical QOL. Given that the 6MWT requires exercise tolerance capacity, aerobic exercise may be helpful in improving QOL in patients undergoing MHD. The GDS-15 score was independently associated with both the PCS and MCS scores, suggesting that depressive symptoms may significantly impact QOL. However, the explanatory power of the MCS model was modest (adjusted R² = 0.1838), indicating that a substantial proportion of the variance in mental QOL remains unexplained. Therefore, depressive symptoms should be interpreted as an important but not exclusive determinant of mental QOL.

A recent report revealed that remote digital health interventions in clinical practice could enhance mental health and contribute to improvements in health-related QOL [15]. Such a digital health intervention delivery approach offers measures to improve mental health and health-related QOL in patients with chronic conditions, including MHD characterized by sedentarism. These findings are consistent with those of an umbrella systematic review from 2023 [16] regarding the effectiveness of physical activity interventions for improving mental health symptoms in adult patients. Physical activity was effective in reducing symptoms of depression and anxiety across all clinical conditions, with a larger effect size observed in people with chronic disease. Regular exercise was correlated with fewer depressive symptoms [16]. A previous report suggested that physical exercise might offer multiple benefits to patients with CKD, and an improvement in QOL was also evident from the KDQOL-36™ survey. Although routine low-intensity physical exercise has no negative effect on renal function, it improves aerobic and functional capacity, positively impacting QOL [17]. Nevertheless, in the present study, physical performance variables were not independently associated with MCS, suggesting that improvements in physical function alone may not be sufficient to enhance mental QOL.

Patients undergoing MHD have a very high mortality risk because of cardiovascular diseases such as chronic heart failure, and sedentary patients undergoing MHD have an even higher mortality risk [18]. This increased risk is due to the combined effects of protein-energy wasting, uremic acidosis, and inflammatory cachexia, which lead to sarcopenia and are aggravated by a sedentary lifestyle. Collectively, these factors result in a progressive downward spiral of deconditioning [19]. Because physical inactivity is a major health problem in patients undergoing MHD, the importance of renal rehabilitation (RR) has been proposed. The frailty measures hospitalization program is designed for patients undergoing MHD who can walk independently. Oral frailty was evident in approximately 70% of patients [8], and motor functions, such as grip and leg muscle strength, walking ability, and SPPB, were already impaired in more than half of this population. Accordingly, in order to detect the reversible state of pre-frailty, it is important that this rehabilitation program, including the assessment of physical and oral frailty and CGA, should be applied to relatively healthy subjects rather than to populations with irreversible frailty. The Japanese Society of Renal Rehabilitation (JSRR) was established in 2011 to promote and disseminate RR in Japan. The Renal Rehabilitation Guideline has been issued, in which three exercises are prescribed: aerobic exercise, resistance exercise, and flexibility exercise [20]. In March 2018, the JSRR established a certification program for Registered Instructors of RR (RIRR) with the aim of improving the quality of RR and educating RR professionals in Japan. Japan leads the field of RR in four ways: it has a national society (JSRR), certification for RIRR, guidelines for RR, and a national health insurance reimbursement program for RR. Additionally, Japan is the only country in the world with a national health insurance system for RR [21]. The exercise therapy proposed in the guidelines for RR is now strongly recommended for patients with CKD [22], because such Japanese rehabilitation has been suggested to improve exercise tolerance, walking ability, and physical QOL.

A limitation of the current study is its restriction to a single institute in Japan. It is well known that variation exists in QOL across countries [3]. Thus, the generalizability of our findings to hemodialysis populations worldwide requires further evaluation. Second, the data were obtained from an observational study, which may have been prone to selection and confounding biases. Therefore, our results should be interpreted with caution. Some authors have suggested that serial assessment is a useful way to monitor the disease course and response to therapy. Moreover, health-related QOL assessments have been shown to improve patient-physician communication. Third, we had limited information on demographic and clinical data that might have been associated with health-related QOL, such as marital status, educational level, socioeconomic status, remaining renal function, or other clinical parameters related to patients’ perceptions of health-related QOL. For patients on hemodialysis, social support is valuable for those who are married, live with family members, and have social support networks. Therefore, it is reasonable to assume that enhancing social support should play a significant role in improving QOL. The role of social support was not evaluated in the current analysis, although higher levels of social support have been found to be associated with higher QOL [23]. Other factors may also lead to bias, including dialysis dose, severe comorbid illnesses, and hemoglobin levels.

IADLs and walking ability were commonly reduced in patients undergoing MHD. The current findings suggest that while RR is important for improving physical QOL, it may not be sufficient to improve mental QOL. Given that depressive symptoms were the sole independent determinant of MCS, additional consideration of psychological factors may be needed when aiming to improve overall QOL in patients undergoing MHD.

## Conclusions

Patients undergoing hemodialysis experience an increased risk of hospitalization and mortality, as well as reduced health-related QOL. The results of the current analysis revealed that physical activity interventions could increase overall QOL by improving walking ability. The GDS-15 score might be associated with improvement in QOL, which is supported by previous reports showing that depressive symptoms were alleviated by physical activity. Therefore, the frailty measures hospitalization program, including physical exercise instruction and mental health screening, might contribute to enhancing the QOL of patients undergoing MHD.

## References

[1] Estoque RC, Togawa T, Ooba M, Gomi K, Nakamura S, Hijioka Y, Kameyama Y (2019). A review of quality of life (QOL) assessments and indicators: towards a "QOL-Climate" assessment framework. Ambio.

[2] Masaki T, Hanabusa N, Abe M (2024). 2023 Annual dialysis data report, JSDT renal data registry. J Jpn Soc Dial Ther.

[3] Brown EA, Zhao J, McCullough K (2021). Burden of kidney disease, health-related quality of life, and employment among patients receiving peritoneal dialysis and in-center hemodialysis: findings from the DOPPS program. Am J Kidney Dis.

[4] Sorensen EP, Sarnak MJ, Tighiouart H (2012). The kidney disease quality of life cognitive function subscale and cognitive performance in maintenance hemodialysis patients. Am J Kidney Dis.

[5] Liu X, Meng J, Zhang S (2025). Analysis of factors influencing quality of life in hemodialysis patients based on KDQOL-36: a cross-sectional study. Int J Artif Organs.

[6] Farivar SS, Cunningham WE, Hays RD (2007). Correlated physical and mental health summary scores for the SF-36 and SF-12 Health Survey, V.I. Health Qual Life Outcomes.

[7] Laucis NC, Hays RD, Bhattacharyya T (2015). Scoring the SF-36 in orthopaedics: a brief guide. J Bone Joint Surg Am.

[8] Ina K, Tenma M, Makino S (2025). Oral frailty and its association with cognitive function and muscle strength in patients on maintenance hemodialysis: a retrospective observational study. Kidney Dial.

[9] Green J, Fukuhara S, Shinzato T (2001). Translation, cultural adaptation, and initial reliability and multitrait testing of the Kidney Disease Quality of Life instrument for use in Japan. Qual Life Res.

[10] Toba K (2005). [The guideline for comprehensive geriatric assessment]. Nihon Ronen Igakkai Zasshi.

[11] Chuasuwan A, Pooripussarakul S, Thakkinstian A, Ingsathit A, Pattanaprateep O (2020). Comparisons of quality of life between patients underwent peritoneal dialysis and hemodialysis: a systematic review and meta-analysis. Health Qual Life Outcomes.

[12] Belafsky PC, Mouadeb DA, Rees CJ, Pryor JC, Postma GN, Allen J, Leonard RJ (2008). Validity and reliability of the Eating Assessment Tool (EAT-10). Ann Otol Rhinol Laryngol.

[13] Cederholm T, Jensen GL, Correia MI (2019). GLIM criteria for the diagnosis of malnutrition - a consensus report from the global clinical nutrition community. Clin Nutr.

[14] Kanda Y (2013). Investigation of the freely available easy-to-use software 'EZR' for medical statistics. Bone Marrow Transplant.

[15] Greenwood SA, Young HML, Briggs J (2024). Evaluating the effect of a digital health intervention to enhance physical activity in people with chronic kidney disease (Kidney BEAM): a multicentre, randomised controlled trial in the UK. Lancet Digit Health.

[16] Tentori F, Elder SJ, Thumma J (2010). Physical exercise among participants in the Dialysis Outcomes and Practice Patterns Study (DOPPS): correlates and associated outcomes. Nephrol Dial Transplant.

[17] Singh B, Olds T, Curtis R (2023). Effectiveness of physical activity interventions for improving depression, anxiety and distress: an overview of systematic reviews. Br J Sports Med.

[18] O'Hare AM, Tawney K, Bacchetti P, Johansen KL (2003). Decreased survival among sedentary patients undergoing dialysis: results from the dialysis morbidity and mortality study wave 2. Am J Kidney Dis.

[19] Kohzuki M (2024). Renal rehabilitation: present and future perspectives. J Clin Med.

[20] Yamagata K, Hoshino J, Sugiyama H (2019). Clinical practice guideline for renal rehabilitation: systematic reviews and recommendations of exercise therapies in patients with kidney diseases. Renal Replacement Therap.

[21] Zelle DM, Klaassen G, van Adrichem E, Bakker SJ, Corpeleijn E, Navis G (2017). Physical inactivity: a risk factor and target for intervention in renal care. Nat Rev Nephrol.

[22] Villanego F, Naranjo J, Vigara LA (2020). Impact of physical exercise in patients with chronic kidney disease: systematic review and meta-analysis. Nefrologia (Engl Ed).

[23] Fuertes JN, Friedman OB, Moore MT, Rubinstein S (2025). CKD patients’ emotional well-being: an examination of their psychological stressors and support factors. Kidney Dial.

